# Accuracy of verbal self-reported blood glucose in teenagers with type I diabetes at diabetes ski camp

**DOI:** 10.1186/2251-6581-13-14

**Published:** 2014-01-08

**Authors:** Matthew Chae, David M Reith, Paul A Tomlinson, Jenny Rayns, Benjamin J Wheeler

**Affiliations:** 1 Department of Women’s and Children’s Health, University of Otago, Dunedin 9045, New Zealand; 2 Department of Women’s and Children’s Health, University of Otago, Invercargill 9812, New Zealand; 3 Department of Endocrinology, Southern District Health Board, Dunedin 9045, New Zealand; 4 Edgar National Centre for Diabetes and Obesity Research, University of Otago, Dunedin 9045, New Zealand

**Keywords:** Type 1 diabetes mellitus, Adolescent, Self-monitoring blood glucose, Self-management

## Abstract

**Background:**

While there have been considerable advances in diabetes management, self-monitoring of blood glucose remains vital. A number of studies, predominantly in adults, have confirmed that logbook entries are prone to a number of common errors. To date, no studies in either adults or children have looked at the accuracy of verbally reported self-monitored blood glucose levels (SMBG). Our aim was to determine the accuracy of verbally reported SMBG levels in adolescents at a diabetes camp.

**Methods:**

Dual Data (verbally reported and meter-downloaded values) were obtained as part of camp safety monitoring from 20 adolescents (aged 13–18 years) attending a 3 day diabetes winter camp. Blood glucose values were classified as: accurate, absent/phantom, or modified - verbally reported value > / < meter downloaded value. No participant had prior awareness of the planned meter data download at camp conclusion.

**Results:**

Discrepancies between verbally reported and meter downloaded values were observed in 14/20 (70%) participants and in 53/394 (13.5%) instances of testing. Absent/Phantom readings were the most common error at 30/394 (7.6%). Errors relating to hypoglycaemia were seen in 8/47 (17%) hypoglycaemia-related incidents of testing. No relationship with HbA1c was found between those with reporting errors and those without (p > 0.05).

**Conclusion:**

While 70% of adolescents had errors, the overall error rate at 13.5% is lower than that previously reported for logbook studies. While this rate is lower than expected, misreporting remains a concern, particularly in the context of diabetes camp and exercise induced hypoglycaemia.

## Introduction

In the last 20 years, the core principles of diabetes care have been influenced by the findings of the Diabetes control and complications trial (DCCT): a landmark study which showed that good metabolic control achieved through an intensified insulin regimen, can delay and/or prevent the onset of diabetes complications [1,2]. These beneficial effects, observed in both the adult and adolescent population, have provided significant reason to encourage tight regulation of glycaemic control [3].

Along with technological and philosophical advances in insulin therapy, glucose monitoring has evolved from the extremes of ‘water tasting’ in the 11^th^ century [4] to the recent and on-going developments in real-time continuous subcutaneous glucose monitoring. Despite these technological developments, self-monitoring of blood glucose (SMBG) remains a cornerstone of modern intensive diabetes management. In the clinic and at home, SMBG can be reported in a number of ways. More recently, software has been available to directly download meters and display this information in a variety of formats. However, traditional techniques of reporting, such as paper log books, remain important. SMBG can also be reported verbally. This is an everyday occurrence in most households with a child or teenager with diabetes, but also occurs during clinician/diabetes team interactions. The accuracy and reliability of verbal and logbook reported SMBG is vital for the safe and effective treatment of T1DM.

While clinicians acknowledge the often inaccurate nature of self-reported SMBG, this is surprisingly understudied. Only a handful of studies have been conducted, with only two touching on adolescence. All have examined logbook reported vs. meter-download readings. In 1984, the first study revealed 26% of self-reported values were not concordant with the meter-downloaded values, and 75% of subjects modified their reports to a significantly lower value [5]. These findings have been confirmed in three additional papers in adults [6-8], and one in an adolescent population [9]. One further study in 19 adolescents explored the reliability of self-reported frequency of daily glucose monitoring. The majority over-reported their frequency of glucose monitoring, but this study did not compare or report any actual blood glucose levels [10]. Therefore to date, despite how important and common these issues are for diabetes management, no studies in children or adults have explored the accuracy of verbally reported values.

The aim of the present study was to test the accuracy of verbally-reported SMBG vs. meter-downloaded values in an adolescent population using routinely collected data and the existing blood glucose safety monitoring protocols of an annual adolescent diabetes winter camp.

## Methods

The paediatric diabetes service at the Southern District Health Board runs an annual diabetes winter camp, at the Coronet Peak ski field, Queenstown, NZ. Adolescents with diabetes were invited, to a maximum of 30. The camp commenced at 1600 Thursday and ended at 1200 Sunday (3 nights), and consisted of various activities such as skiing, rock climbing, mini golf, and ice skating. Camp protocol required the participants to self-monitor blood glucose, and verbally report results to staff at various compulsory time points: pre-prandial (pre-breakfast, pre-morning tea, pre-lunch, pre-afternoon tea, pre-dinner and pre-supper/bedtime) and overnight (1-2 am). These, along with exact time taken, were documented in a log book format by three staff members. Approximately 18 verbally-reported readings were expected per participant. Additionally, staff carried an individually labelled and numbered glucose meter to be used when necessary, with any use of this meter clearly identified in the log book, for later exclusion from comparison.

For the purposes of this study, at completion of camp, the participant’s meters were downloaded via their respective software (Co-pilot™ and Accu-Chek360°™) to obtain all values and times of tests performed during the camp. The date and time of all glucose meters used on camp were checked, adjusted, and synchronised prior to the first compulsory SMBG, and the correct time confirmed again prior to meter download. Meters have never been downloaded on camp before, and no participant had prior awareness of the planned download.

### Management of Hypoglycaemia and hyperglycaemia

Hypoglycaemia was defined as any value <4.0 mmol/l and was required to be verbally reported to staff to be treated appropriately, and then documented. 10-20 g of fast acting glucose was given, and a retest of blood glucose was to be done at 10 minutes and verbally-reported. Once values were above 4.0 mmol/L, a snack, of 10-20 grams slow acting carbohydrate was given, however if BGLs remained below 4.0 mmol/l the cycle was repeated again, including compulsory SMBG reporting and staff documentation. If any concern regarding the safety of participants was raised, a hospital meter was to be used to confirm blood glucose levels.

### Analysis

At completion of camp, staff log books of all verbally reported SMBG were compared with their corresponding meter downloads (including any corresponding hospital meter downloads), with particular attention made to exact date, time and value. Discrepancies between verbally-reported and meter-downloaded value were categorised into: 1) Absent test/Phantom values - verbally reported value with no actual test done; 2) Modified - verbally reported BGL > Meter downloaded BGL 3) Modified - verbally reported BGL < Meter downloaded BGL; 4) Accurate (minor rounding discrepancies to/within a whole number were permitted e.g. 20.7 mmol/L verbally reported as 20 mmol/L, as long as the rounding did not alter the status regarding hypoglycaemia. Any discordance/errors related to hypoglycaemia were also analysed using the above categories. Between group comparisons for continuous variables were performed using the t-test in Stata 9.0 (College Station, TX, USA). In addition, a descriptive analysis was done to distinguish the age, sex, duration of diabetes and HbA1c levels of those with discordant data vs. those with concordant data. The closest HbA1c clinic reading within 2 months of the camp was obtained from the Dunedin Hospital electronic diabetes database along with baseline demographics. HbA1c was measured using DCA Vantage Analyzer (Siemans) which has acceptable levels of accuracy and reliability [11,12].

### Ethics

This study initially commenced as a clinical audit of BGLs on diabetes winter camp. Downloading of meters has never occurred on camp before. However, it is an integral aspect of current clinic practice so was not seen as a unique or novel tool. Following review of the obtained BGL data, unique data of value to the wider clinical/diabetes community was noted. At this point, as intentions had expanded from simple clinical audit to presentation to a wider audience, ethical approval was sort. This was important, as when viewed with this new purpose in mind, the study design was concealed from all participants. Retrospective ethical approval for the study was obtained from the University of Otago ethics committee (reference number 12/316, in accordance with the Declaration of Helsinki) on condition that retrospective consent to analyse and publish data was obtained from all participants and their families.

## Results

Excluding those with non-T1DM, a total of 22 Adolescents aged 13–18 (mean 14.7 years) attended the 2012 winter camp, of which an even split of gender was observed. Dual Data (verbally reported and meter-downloaded values) was obtained for 20 out of the eligible 22 (91%) camp participants. Data was not obtained in two due to technical failure with meter download. Overall, 412 individual occasions of potentially reported SMBG were identified. A further 18 recordings were excluded from analysis: verbally reported data was missing on 13 occasions; and hospital meters were used to measure blood glucose levels on 5 occasions. The total data available for comparison (verbal reporting vs. meter download) was 394 episodes. Baseline characteristics are shown in Table 1.

**Table 1:** Baseline demographics

	**Total sample (N = 20)**
Female Sex; n (%)	10 (50%)
Age^a^ (yr)	14.7 (13–18)
Diabetes duration^b^ (months)	39 (10–157)
Insulin Regimen MDI: CSII (n)	17:3
Latest HbA1c^a^; mmol/mol (%)	81 (9.5%); (55–130)
Number of verbal BGL recorded per individual^c^	20 ± 2.9†
Number of meter BGL recorded per individual^c^	18 ± 4.4†

^a^expressed as mean (range); ^b^expressed as median (range); ^c^expressed as mean ± SD; †p < 0.05 (t-test) for number of verbal vs. meter records per individual.

MDI – Multiple daily injections; CSII – continuous subcutaneous insulin infusion; BGL – Blood glucose level.

Significant errors between verbally reported and meter downloaded values were observed in 14/20 (70%) participants or in 53/394 (13.5%) instances of testing. If minor rounding discrepancies were also included 101/394 (25.6%) discordant readings were seen. However 39/53 (73.6%) of total errors were seen in 5/20 participants. The errors/discordant readings broken down by type are shown in Table 2.

**Table 2:** Classification of reporting errors

**Error type**	**Number (%)**
Testing Absent/Phantom reading	30/394 (7.6%)
Reported BGL > meter BGL	5/394 (1.3%)
Reported BGL < meter BGL	18/394 (4.6%)
Total Discordance/Errors	53/394 (13.5%)

Errors relating to misclassification of hypoglycaemia were seen 8/394 (2%) tests, or 8/47 (17%) hypoglycaemia related incidents of testing. Hypoglycaemia related errors occurred in 6/20 (30%) individuals.

Table 3 shows the characteristics of participants comparing those with reporting errors vs. no reported errors. While HbA1c was higher in the subjects with reported errors this was not statistically significant (all p values >0.05). Additionally, no relationship with HbAa1c was found when analysed for the five participants responsible for 73% of errors.

**Table 3:** Subject characteristics – any error vs. no errors

	**Reporting errors**			**No reported errors**		
	**14**			**6**		
Subjects (N)	Male	Female	Total	Male	Female	total
	7	7	14	3	3	6
Age mean, years (range)	14.4	14.9	14.6	15.3	14.3	14.8
	(13 – 15)	(13 – 18)	(13 – 18)	(14 – 17)	(13 – 15)	(13 – 17)
HbA1c mean, mmol/mol (%)	80 (9.5%)	87 (10.1%)	83 (9.3%)	70 (8.6%)	82 (9.7%)	76 (9.1%)
HbA1c range, mmol/mol (%)	70 – 93	55 – 130	55 – 130	61 – 82	79 – 88	61 – 88
	(8.6 – 10.7)	(7.2 – 14)	(7.2 – 14)	(7.7 – 9.6)	(9.4 – 10.2)	(7.7 – 10.2)
Diabetes duration median, months (range)	40	50	45	14	56	42
	(10 – 157)	(12 – 85)	(10 – 157)	(10 – 51)	(32 – 58)	(10 – 58)

No statistically significant relationships between those with and without reporting errors were seen (independent t-test, all p values >0.05).

## Discussion

This is the first study to test the accuracy of verbal SMBG, finding an overall error/discordance rate of 13.5%, spread over 70% of participants. Quantifying and understanding the error rate for verbally reported SMBG is important, as in the home, clinic or camp setting, verbally self-reported blood glucose values often serve as a proxy to meter values.

While 70% of adolescents had errors, the overall error/discordance rate for verbal SMBG at 13.5% is lower than for most logbook studies, which range from 13.2-50% [5,7,9]. The reason for this is unknown. Verbal reporting is a direct personal interaction compared to the private experience of keeping a log book. We speculate that this, particularly when combined with direct interaction with diabetes professionals, adds a barrier to the fabrication of results. Whether or not this error rate changes in the home setting, or during adolescent to parent interaction, remains unknown.

The majority of errors/fabrications were of the absent/phantom subclass. This error pattern is consistent with previous reports [5,8]. However, at 7.6% of total recordings this is substantially lower than previous logbook studies which found phantom/absent rates of up to 44% [8,9]. If a frequent event, phantom readings have a number of potential consequences: they may have implications for overall glycaemic control, as more frequent testing has been linked to lower HbA1c values [13]; additionally, fabrication of results may cause inappropriate insulin adjustments, which may be harmful. This is particularly the case during periods of prolonged and vigorous exercise, such as in the ski camp setting.

It is concerning that 17% of hypoglycaemia episodes experienced during camp were reported incorrectly. Incorrectly reporting a normal blood glucose as opposed to the actual hypoglycaemic reading was the most common error seen. There are a number of possible explanations for this. It may be in an effort to present a more positive profile of BGLs [7]; and/or to reduce time away from the enjoyable social interaction and activities of camp. Both phantom and incorrectly reporting a normal value as hypoglycaemia were also seen. We speculate the later was used as a strategy to access fast acting carbohydrate in the form of sweets. Whatever the reason, hypoglycaemia remains a much feared acute complication of T1DM, with frequent and accurate SMBG crucial for safe detection and treatment. Again, this is even more important in the context of exercise.

Meter-downloading may have a role in improving safety at diabetes camp. Patient awareness of a future meter download has been shown to dramatically improve the accuracy of log book records in adults [6,8]. If true for verbal reporting, this property could be utilised to ensure more accurate and therefore safer interpretation of verbally reported BGLs at future camps.

While small, and of short duration, with 20 participants, this is one of the largest studies conducted looking at accuracy of SMBG in any context. Data collection was restricted to a diabetes winter camp, which may limit generalizability of our findings. As this is the first study to look at verbal self-reported blood glucose, further studies are needed to confirm and explore these findings across multiple settings and environments (e.g. camp vs. home; reporting to a parent vs. the medical diabetes team). As BGLs were verbally reported following self-monitoring, recall bias could also be a factor influencing results. However, it is unlikely that this explains the majority of errors seen, which were absent test/phantom values, and unlikely to be significantly impacted by errors in recall. Attempts were also made to minimise this with minor rounding errors excluded from analysis. In this study, no relationship between reporting errors and poorer overall glycaemic control (as measured by HbA1c) was seen. While the sample size may limit our power to detect a relationship, this finding is consistent with previous log book studies [5,7]. Additionally, this study was not designed to study the reasons for/motivations driving these inaccurately reported values. Future studies exploring this are needed to fully understand this important phenomenon.

In conclusion, this is the first study to look at the accuracy of verbal self-reported blood glucose in T1DM, finding errors in 70% of participants, with an overall error rate of 13.5%. This rate is considerably lower than that seen in previous log book studies in adults and adolescents. Although low, misreporting remains a concern, particularly in the context of exercise-induced hypoglycaemia. As with other studies, we have not discovered any factors that assist in predicting those at risk of reporting errors. Further study is needed to explore this important and overlooked, every-day aspect of diabetes management.

## Competing interests

The authors declare that they have no competing of interests.

## Authors’ contributions

BW conceived and supervised the research. MC wrote the first draft of the manuscript with assistance from BW. MC, JR and PT managed the patients and protocol while on camp. MC, BW and DR conducted the statistical analysis. All authors contributed to writing and editing the manuscript. All authors read and approved the final manuscript.
